# Supplementary figures and images for: Structural basis for the interaction of human herpesvirus 6B tetrameric glycoprotein complex with the cellular receptor, human CD134
Source: PLoS Pathog. 2020 Jul 17;16(7):e1008648. doi: 10.1371/journal.ppat.1008648 (PMC7367449; doi:10.1371/journal.ppat.1008648)

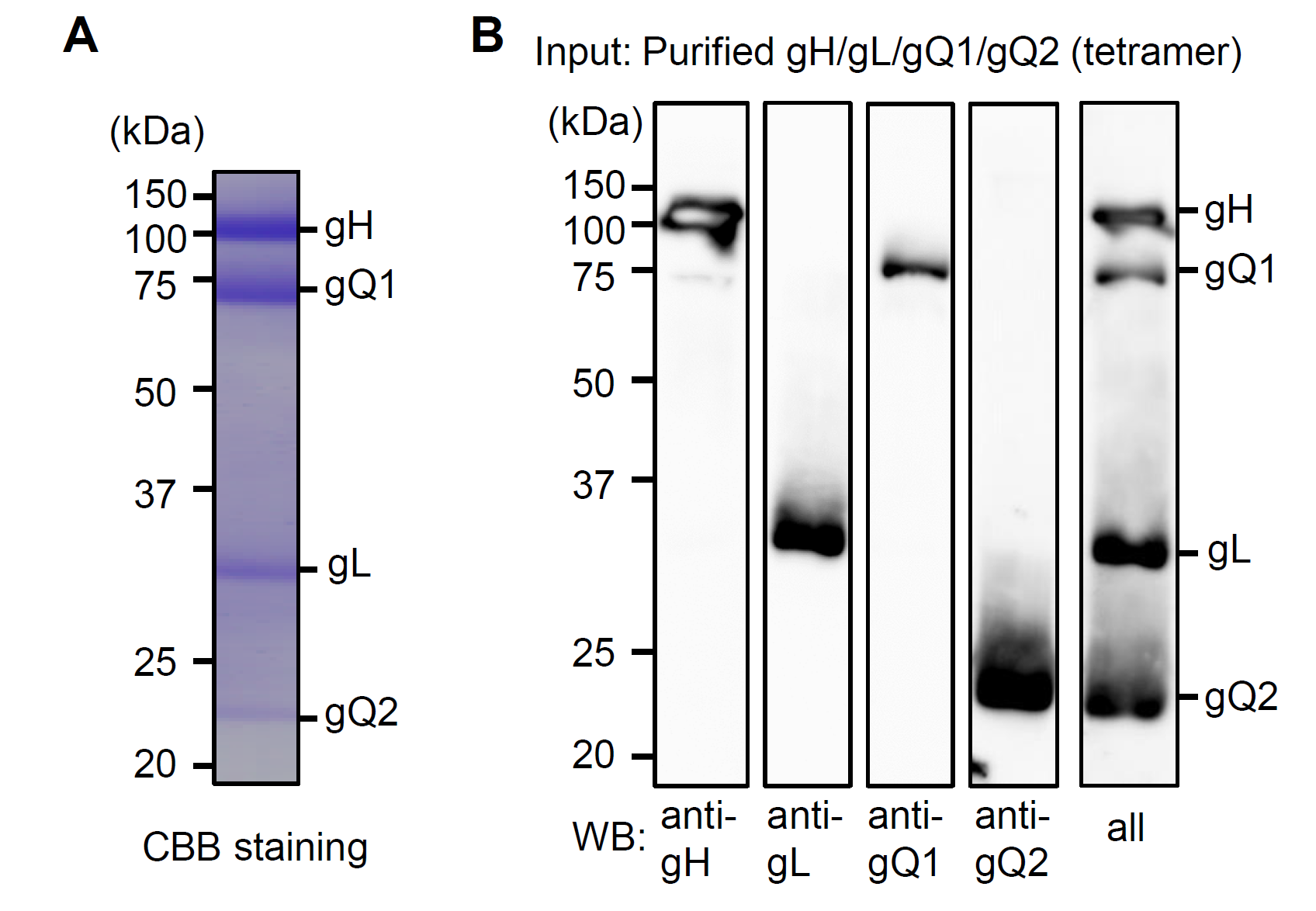

Supplement: S1 Fig — The tetramer prepared by a mammalian expression system and column chromatography was subjected to the SDS-PAGE analysis and detected by CBB staining (A) and Western blotting (B) with Mabs specific to each component. (TIF) [file ppat.1008648.s001.tif]

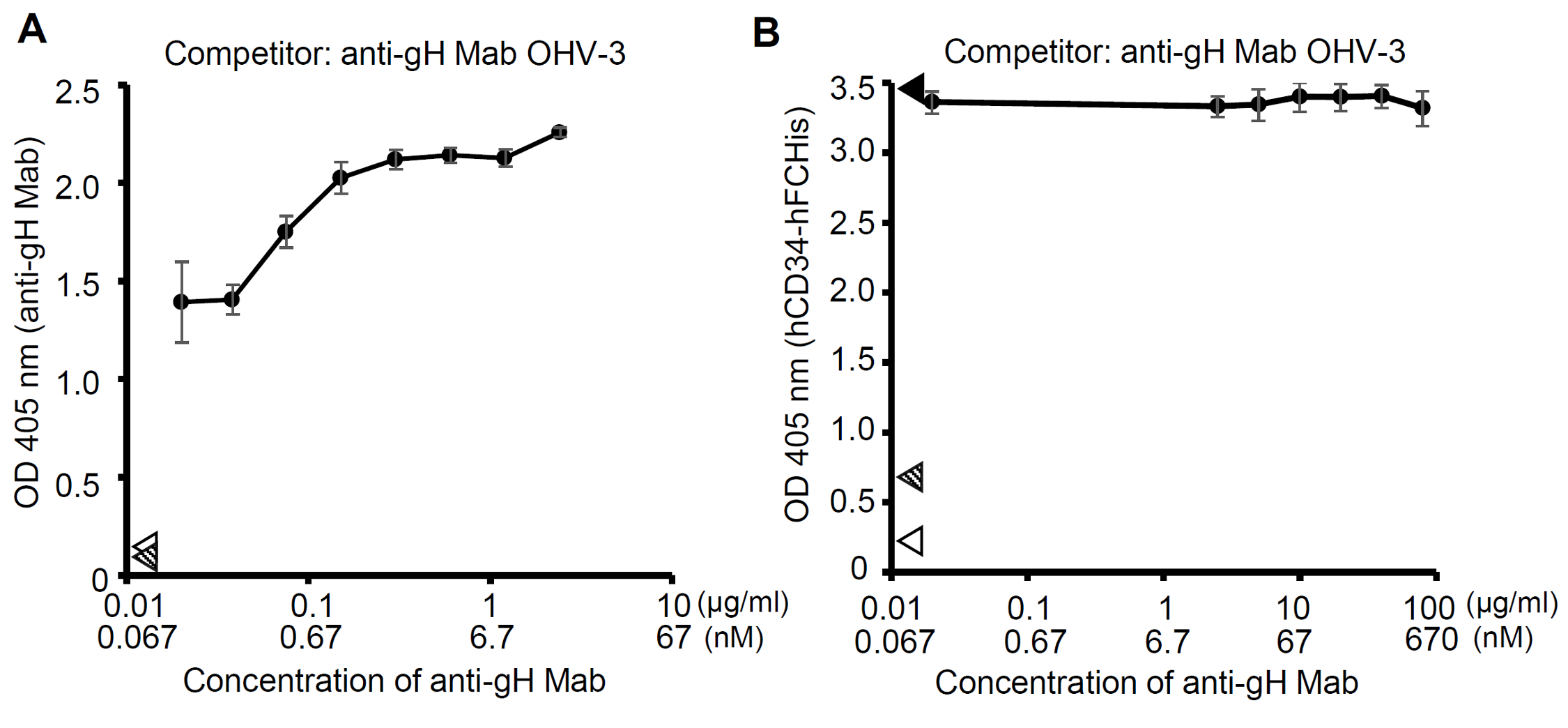

Supplement: S2 Fig — (A) The binding of anti-gH Mab was detected by anti-mouse IgG-HRP instead of anti-human IgG-HRP in the same condition as shown in Fig 2B. The open and striped triangles indicate the value without anti-gH Mab and the value without tetramer in presence of anti-gH Mab at 2.4 μg/ml, respectively. The plotted points are the averages of three wells in the same condition. Bars: SD of the wells. One of the duplicated results is shown. (B) The binding of hCD134-hFcHis to the tetramer was detected by the same ELISA experiment as shown in Fig 2B, in a higher concentration range of anti-gH Mab OHV-3. The concentration of the hCD134-hFcHis was 0.1 μg/ml, and the Mab concentration was varied at 0.02, 2.5, 5.0, 20, 40, and 80 μg/ml as the final concentration. The plotted points are the averages of four wells in the same condition. Bars: SD of the wells. The filled arrowheads and open arrowheads indicate the value without Mab and that without hCD134-hFcHis, respectively. The striped arrow head indicated the value for the hCD134-hFcHis binding in the presence of anti-gQ1 Mab KH-1 at 2.4 μg/ml. One of the duplicate results is shown. (TIF) [file ppat.1008648.s002.tif]

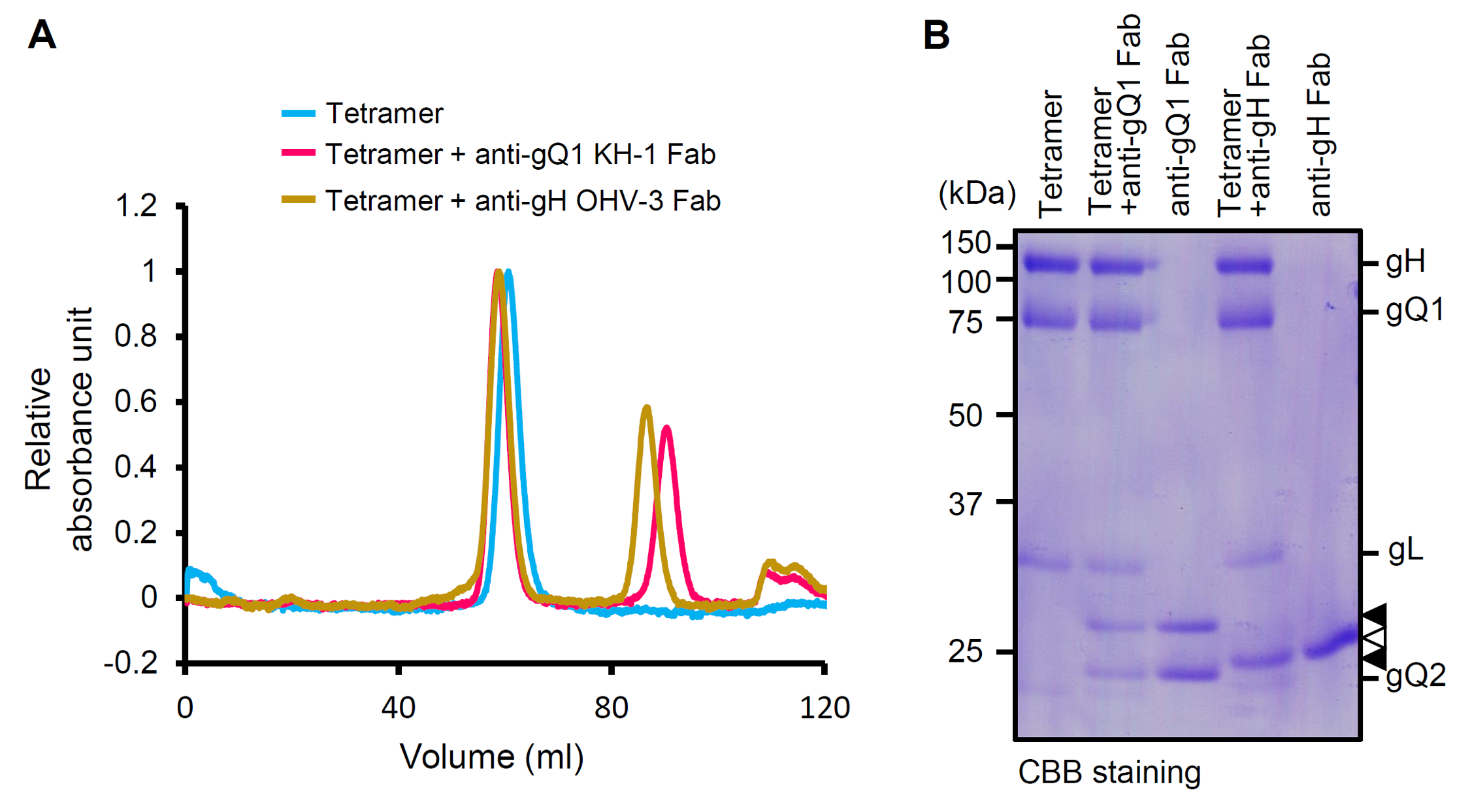

Supplement: S3 Fig — (A) The full chart of the size-exclusion column chromatography experiments are shown. The region from the elution volume 40 ml to 80 ml is shown in Fig 5A. (B) SDS-PAGE analysis of each peak in the panel (A). The band corresponds to the gH, gL, gQ1 and gQ2 are indicated. The bands for Fabs of anti-gQ1 Mab KH-1 and anti-gH Mab OHV-3 are also indicated by filled arrowhead and open allowhead, respectively. (TIF) [file ppat.1008648.s003.tif]
