# Supplementary material for: Structural basis for the interaction of human herpesvirus 6B tetrameric glycoprotein complex with the cellular receptor, human CD134
Source: PLoS Pathog. 2020 Jul 17;16(7):e1008648. doi: 10.1371/journal.ppat.1008648 (PMC7367449; doi:10.1371/journal.ppat.1008648)
Supplement: Attachment — Submitted filename: 200501 Response to reviewers comments.docx [file ppat.1008648.s005.docx]

Responses to Reviewers’ Questions

**Part I - Summary**
Please use this section to discuss strengths/weaknesses of study, novelty/significance, general execution and scholarship.

Reviewer #1: This work provides the first structural depiction of the HHV6 gHgLgQ1gQ2 tetramer, a complex that is required for HHV6 entry into cells. The authors characterize the tetramer binding to receptor and to neutralizing antibodies (nMAbs) and report the EM structure of the tetramer, with or without nMAbs bound.

The authors purified soluble tetramer expressed in mammalian cells (Fig S1). They used SPR to examine tetramer binding to two nMAbs and receptor (Fig 1, Tbl 1). Tetramer affinity for all three was high and, interestingly, the receptor binding had a fast association and dissociation. Using competition ELISA, the anti-gQ1 nMAb was shown to block tetramer-receptor binding, whereas anti-gH nMAb did not (Fig 2), indicating that the two nMAbs employ distinct mechanisms of neutralization. The crystal structures of unliganded Fabs for both nMAbs were solved (Fig 3, Tbl 2). Single particle EM analysis of the tetramer revealed an elongated curved structure with “palm” and “stalk” sites (Fig 4). Manually fitting an gHgL model (based on HCMV gHgL) into the density placed gH in the stalk and gL near the palm. Most of the palm was left unfilled, suggesting that gQ1 and gQ2 reside in the palm. Complexes of tetramer plus each Fab were purified (Fig S2). Single particle EM analysis of these Fab-tetramer complexes showed two distinct sites of Fab binding and supported the earlier manual fitting of the gHgL structure in the density (Fig 5). The anti-gQ1 Fab binding was at the end of the structure, whereas the anti-gH Fab bound to the side the structure.

The work is convincing and logical. The paper is well written and concise. This work will have significant impact in the field of herpesvirus entry into cells. The biochemical (ELISA and SPR) and structural (crystallography and EM) approaches provide strong evidence that receptor binding occurs at gQ1, at the distal end of the tetramer. By following up their fitting of a gHgL model into their tetramer EM density with binding studies of the two Fabs, the work is persuasive that this fitting is accurate. gQ1 and gQ2 appear to dock at the gL tip of gHgL, similar to the location of gO in HCMV trimer and UL128/UL130/UL131 in HCMV pentamer. Thus, this work demonstrates that gHgL complexes from both betaherpesviruses are similar to one another, but quite different than the EBV gHgLgp42 complex, in which gp42 binds along the side of gH. Identifying the structural location of the anti-gH Fab binding is of particular interest because the mode of neutralization for this Fab is unknown (and not due to interference with receptor binding).

Reviewer #2: Nishimura et al. report the modelled structure of the HHV-6B gH/gL/gQ1/gQ2 (tetramer) complex alone and complexed with the Fab fragments of two monoclonal antibodies to gQ1 and gQ2. The authors also determined the affinity of the two mAbs as well as of the CD134 receptor for the tetramer and demonstrate direct inhibition of tetramer binding by the gQ1 antibody KH-1 but not by the gH antibody OHV-3. Using negative-staining EM, averaging and 3D modelling, the authors provide an informative structure of the tetramer complex more or less at the domain level and of the tetramer complexed with each of the two Fabs, fitting the crystal structure of the two Fabs, which they also determined, into the EM model.

The manuscript is generally well written.

The crystal structures of the Fab domains of the variable antibodies provide potentially useful information for further targeted development and optimization of these antibodies.

The overall shape of the tetramer complex is highly interesting to the field.

One question is obvious: Why was the same EM analysis not attempted in complex with CD134, in addition to the Fab fragments? This would have been even more informative.

Response:

A preliminary trial of EM analysis on the tetramer-hCD134 complex did not show any apparent density for the hCD134. We considered that the relatively small size (approximately 20 kDa calculated from the amino acids sequence) and stick-like shape of the hCD134 molecule (Compaan and Hymowitz, *Structure*, 2006) might make it difficult to visualize the density in the resolution of negative stain EM analysis. Moreover, the negative staining method might damage the complex as is generally known, considering the relatively unstable nature of the complex indicated by the rapid dissociation parameter in the SPR analysis (Fig 1C and Table 1). Because of these possible difficulties in sample and experimental technique, we could not include negative stain EM analysis on the tetramer-hCD134 complex in this research. We believe that the cryoelectron microscopy, which is greatly advanced in recent years, or the X-ray crystallography are thought to be much more suitable to reveal the tetramer-hCD134 complex, and thus we are going to pursue the issue in future studies. We noted these information in the Discussion section (Lines 446-455).

**Part II – Major Issues: Key Experiments Required for Acceptance**
Please use this section to detail the key new experiments or modifications of existing experiments that should be absolutely required to validate study conclusions.

Generally, there should be no more than 3 such required experiments or major modifications for a "Major Revision" recommendation. If more than 3 experiments are necessary to validate the study conclusions, then you are encouraged to recommend "Reject".

Reviewer #1: (No Response)

Reviewer #2: As the structural models for the tetramer built in this paper are not illustrative but constitute basically the main piece of novel information contributed by this study, apart from the measured affinities, these models should be deposited in a public database or at least be made available as supplemental information so that the community can use them for visualization or for the informed design of experiments.

Response:

According to the comment, we prepared a Supporting information S1 Data which includes the 3D EM density for the tetramer fitted by the HHV-6B gH/gL model, the 3D EM density for the tetramer + anti-gQ1 Mab KH-1 Fab fitted by the gH/gL model and the Fab structure, and the 3D EM density for the tetramer + anti-gH Mab OHV-3 Fab fitted by gH/gL model and the Fab structure. The legend was shown at the Lines 670-680. The data was cited in the Materials and Methods in the revised manuscript (Lines 629-631).

Line 154 (Wang et al. manuscript submitted) I am not sure what the regulations on these types of citations are. If it is not a preprint, can it be cited? Also what type of manuscript is this, is there some sort of redundancy between the manuscripts? I personally don’t like citations to something that can’t be read - so if it is cited, it should be made available to the editor and the reviewers. Publication as a preprint would be an option, too.

Response:

We agreed with your comment that we should make the cited information available for readers. We provide the additional information about the tetramer preparation in the Materials and Methods section (Lines 513-532).

Fig 2. It would be highly interesting for the reader to see how the inhibitory concentrations in the ELISA assay compare to the inhibitory concentrations in infection experiments for the anti-gQ1 antibdoy KH-1. Aaccording to the 2019 Wang paper, the IC50 for the gQ mAb is 0.17µg/ml, for the gH mAb ~40fold higher at 7.7 µg/ml. The inhibition curve here for KH-1 indicates that IC50 for inhibition of binding of CD134 is higher than for infection. While the concentration range around the IC50 in infection assays is tested for the gQ1 mAb KH-1, it is not tested for the gH mAb OHV-3 in this competition assay. It could therefore be that OHV-3 also inhibits CD134 binding via an allosteric effect on the tetramer. Even if unlikely, this can’t be ruled out because the concentration range where an inhibitory effect on infection was observed is not reached in the experiment. The experiment should be repeated using higher concentrations of OHV-3, or the possibility of allosteric inhibition should at least be discussed. It may seem unlikely, as the affinity as measured by biacore of OHV-3 was higher than that of KH-1. But affinity by biacore, affinity on the virus surface, and affinities on ELISA plates, as well as IC50 in infections may not be directly comparable. These possibilites should be discussed in lines 417-431, line 451. Alternatively, experimental evidence of an OHV-3-tetramer-CD134 complex e.g. by immunoprecipitation of tetramer-CD134 complex would support the authors’ interpretation and rule out allosteric inhibition of receptor binding, and would clarify the mode of action of OHV-3. This is actually an easy experiment and would strongly support the author’s conclusions. Or maybe this has already been done (not to my knowledge) and could be cited?

Response:

According to the reviewer’s suggestion, we performed an ELISA experiment to assess the competition between the receptor hCD134 and the anti-gH Mab OHV-3 for binding to the tetramer at a higher concentration range of the Mab. The anti-gH Mab OHV-3 had no influence on the tetramer-hCD134-hFcHis interaction even at the highest concentration, that is, 80 µg/ml (new S2B Fig). We also added the data for anti-gH Mab OHV-3 binding to the tetramer in presence of the hCD134-hFcHis, and it was demonstrated that the anti-gH Mab bound to the tetramer (new S2A Fig). Thus, we concluded that the anti-gH Mab OHV-3 has no inhibitory activity on the receptor binding at least in our vitro experiment with purified proteins. We presented these data in the Results section (Line 210-216) with the new S2 Fig and the legend (Lines 646-661), and also added discussion about the possibility of allosteric effect, and the difference between this experiment and previous infection assay (Lines 469-473).

Line 288: please explain “some of the 2D averaged…”. What percentage? Could this be an entirely different conformation? If this “spiral shape” is mentioned, it should be illustrated properly – which of the micrographs displays this shape?

Response:

We should correct the overstatement about the “spiral shape” because it was just an impression from the 2D images and was not evident in this study. Thus, we deleted all the related descriptions in the paragraph (Lines 293-301).

**Part III – Minor Issues: Editorial and Data Presentation Modifications**
Please use this section for editorial suggestions as well as relatively minor modifications of existing data that would enhance clarity.

Reviewer #1: - The authors describe a spiral structure (line 228) observed in the 2D EM images of the tetramer, but they do not show a picture of that structure. They could consider adding a sample spiral image to Fig. 4 (or as a supplemental figure), in case this structure is of interest to others in future work.

Response:

As explained in the response to the last comment from reviewer #2 in the Major Issue, the spiral shape was not evident and it was our overstatement. Thus, we deleted all the related descriptions in the corresponding paragraph (Lines 293-301).

- The authors describe that interpretation of the 2D images for the anti-gH Fab was more difficult than for the anti-gQ1 Fab (line 342). They could expand on this in a sentence to explain why.

Response:

The interpretation of the 2D images for the complex with anti-gH Mab OHV-3 Fab was relatively difficult because the Stalk, Palm and the Fab density could not be distinguished from each other in the 2D images with multiple knobs. We added this explanation at the corresponding part (Lines 345-347).

- In Fig 1, the authors could label figure panels with the analyte and immobilized molecule. The information is in the figure legend, but labeling the graphs would be helpful for quick comprehension. Part D x axis could read “concentration of tetramer (nM)”.

Response:

According to the comment, we added the labels for analyte and ligand (immobilized molecule) in the Fig 1A, 1B and 1C. We also revised the x axis label of Fig. 1D to ”Concentration of tetramer (nM)” as suggested.

- In Fig 2, the authors could add names of Abs to graphs so reader doesn’t have to refer to the legend to understand the result immediately.

Response:

According to the comment, we added labels for panels to indicate the competitors in Fig 2A and 2B.

- In Fig 3, the green dotted line isn’t clear to see, but this isn't critical.

Response:

We changed the green dots to arrows colored by light green in the Fig 3D and 3E.

- In Fig 5, labeling the figure with the names of the Fabs would save the reader time. Coloring gHgL in the density would help distinguish it from the Fab, especially in part E.

Response:

According to the reviewer’s suggestion, we added the sample labels in Fig 5B, 5C, 5D, and 5E. To distinguish the gH/gL and the Fab, we colored the density around each Fab by gray in Fig 5C, 5E and Fig 6.

Reviewer #2: Line 39: please explain “significantly high” or rephrase.

Response:

We rephrased that simply as “high” at the Line 37.

Fig 2.: The concentrations of the antibodies should also be given in nM, for better comparison to figure 1.

Response:
We added the line for nM under that for µg/ml in the Fig 2 and the new S2 Fig. We used the molecular weight for mouse IgG 150,000 g/mol to translate the antibody concentration µg/ml to nM. For example, 10 µg/ml was translated to 67 nM.

Line 398-402: I am not sure that I read this sentence correctly, and I am not sure that I understand what exactly it means. It should be rephrased. Also, I think that a figure would be much more helpful to illustrate the similarities and differences between the different gH/gL/… complexes. Again, the models should be made available for viewing in a molecule viewer, which would make comparisons easier.

Response:

We meant that the EBV gH/gL/gp42 did not have the gp42 on top of gL but it associates with the gH apart from gL. We rephrased the corresponding part in the Discussion (Lines 403-405) with additional reference (Reference No.38) which contains the relevant figure. We also provided the 3D data for readers as the Supporting information S1 data.

Line 411-414: I understand the Akkapeiboon reference in a way that there is experimental evidence for a gL-gQ2 disulfide bond for HHV-6A, so that is not exactly a prediction, but rather fits with the data for HHV-6A.

Response:

As pointed out in this comment, the data is for HHV-6A tetramer. We revised the corresponding sentence in the Line 416-418.
